# Competencies to promote collaboration between primary and secondary care doctors: an integrative review

**DOI:** 10.1186/s12875-020-01234-6

**Published:** 2020-09-02

**Authors:** Marijn Janssen, Margaretha H. Sagasser, Cornelia R. M. G. Fluit, Willem J. J. Assendelft, Jacqueline de Graaf, Nynke D. Scherpbier

**Affiliations:** 1 Department of internal medicine Nijmegen, Radboud university medical centre, Geert Grooteplein Zuid 10, postal route 463, PO box 9101, 6500 HB Nijmegen, the Netherlands; 2 Network of GP Specialty Training Institute in The Netherlands, Utrecht, the Netherlands; 3 Radboud university medical centre, Radboudumc Health Academy, Nijmegen, the Netherlands; 4 Department of Primary and Community Care, Radboud university medical centre, Radboud Institute for Health Sciences, Nijmegen, the Netherlands

**Keywords:** (intraprofessional) collaboration, Primary-secondary care interface, Doctors, Competencies, Integrative review

## Abstract

**Background:**

In a society where ageing of the population and the increasing prevalence of long-term conditions are major issues, collaboration between primary and secondary care is essential to provide continuous, patient-centred care. Doctors play an essential role at the primary-secondary care interface in realising ‘seamless’ care. Therefore, they should possess collaborative competencies. However, knowledge about these collaborative competencies is scarce. In this review we explore what competencies doctors need to promote collaboration between doctors at the primary-secondary care interface.

**Methods:**

We conducted an integrative literature review. After a systematic search 44 articles were included in the review. They were analysed using a thematic analysis approach.

**Results:**

We identified six themes regarding collaborative competencies: ‘patient-centred care: a common concern’, ‘roles and responsibilities’, ‘mutual knowledge and understanding’, ‘collaborative attitude and respect’, ‘communication’ and ‘leadership’. In every theme we specified components of knowledge, skills and attitudes as found in the reviewed literature. The results show that doctors play an important role, not only in the way they collaborate in individual patient care, but also in how they help shaping organisational preconditions for collaboration.

**Conclusions:**

This review provides an integrative view on competencies necessary for collaborative practice at the primary-secondary care interface. They are part of several domains, showing the complexity of collaboration. The information gathered in this review can support doctors to enhance and learn collaboration in daily practice and can be used in educational programmes in all stages of medical education.

## Background

In a society where ageing of the population and the increasing prevalence of long-term conditions are major issues, collaboration between primary and secondary care is essential. A substantial part of the patient population has more than one chronic condition, leading to many transitions between primary and secondary care. These transitions are associated with a high risk of medical errors [1, 2]. To assure continuous, patient-centred care, health systems should be seen in terms of their interrelations, instead of fragmented care with ‘professional silos’ [3].

Primary care plays an important role in the care and the coordination of care for patients with multimorbidity [4]. To successfully manage these patients, primary care providers need easy access to specialised knowledge from secondary care [5]. At the level of secondary care, specialists face more hospitalisations of patients with multimorbidity and are surrounded by an increasing amount of diagnostic and therapeutic possibilities. In order to make good decisions about diagnoses and treatments, they need access to patient-related knowledge from primary care and need to have insight into what primary care has to offer to these patients.

To provide good collaboration, both primary and secondary care doctors should be equipped with collaborative competencies. Doctors’ training programmes acknowledge the importance of learning collaboration; it is seen as a core competency in postgraduate and continuing medical education, with increasing attention for the primary-secondary care interface [6, 7]. Nevertheless, various questions are still unanswered: What does good collaboration mean? What makes a doctor a good collaborator? What should they do or know? Competency frameworks can help answer these questions and provide a common lens through which professionals can understand, describe and implement collaborative practice [8–11]. To our knowledge, such frameworks do not exist for collaboration between primary and secondary care doctors.

To fill this knowledge gap, we performed a literature review aiming to answer the question: “Which competencies do doctors need to promote collaboration between primary and secondary care doctors, in order to provide good patient care at the primary-secondary care interface?” This knowledge is essential when developing education in the collaborative competency domain, in graduate as well as in postgraduate training. Moreover, with this knowledge doctors can recognise opportunities to improve theirs and other’s collaborative competencies to optimise continuous, patient centred care.

## Methods

Since an integrative review approach is the the broadest type of review methods allowing for the simultaneous inclusion of experimental and non-experimental research in order to more fully understand a phenomenon of concern [12], we considered this the most suitable method to answer our research question.

The research team consisted of two general practitioners (GPs) (NS and WA), one medical specialist (JG), one medical specialty resident and PhD student in the field of intraprofessional collaboration (MJ), one educationalist and medical doctor (CF) and one educationalist (MS). The GPs and the medical specialist were involved in (post)graduate training programmes.

### Search strategy

We performed a literature search with the following search terms and their related terms: (1)“interprofessional or interdisciplinary collaboration”, (2)“primary care doctors”, (3)“secondary care doctors” and (4)“competencies (or skills, or knowledge, or attitude)”. We used both MESH-terms and free-text terms, a full list of the search terms is available online (additional file 1). We searched the databases MEDLINE, CINAHL, Psychinfo and ERIC. The search included literature between 1960 till April 2019. Title and abstracts, if available, were screened by the first author (MJ). When relevant, we screened references or reversed citations. Because of the high number of citations of articles retrieved from the Journal of Interprofessional Care we additionally hand searched all editions of this journal from 1992 until issue 2 of 2019.

### Inclusion

After screening of title and abstracts, selected articles were read full text by the first author (MJ) and a second researcher (NS or MS) to determine their inclusion. An article was included when it discussed collaboration between doctors from both primary and secondary care, and described competencies associated with this collaboration (see Table 1 [13–15]). Articles discussing collaboration between doctors in either primary or secondary care or collaboration between doctors and other health professionals were excluded. Because we aimed to capture the full depth and breadth of the topic, we did not limit on type of publication [12].

**Table 1:** Definitions of terms used in the inclusion criteria

Terms in inclusion criteria	Definition used
Collaboration	**The action of working with someone to produce something.**
Primary care doctor	A doctor who treats all common medical conditions and refers patients to hospitals and other medical services for urgent and specialist treatment. The doctor focuses on the health of the whole person combining physical, psychological and social aspects of care.
Secondary care doctors	Doctors providing emergency care or planned medical care upon referral by another (primary) care professional.
Competency	An ability of a health professional, integrating multiple components such as knowledge, skills, values and attitudes.

To define if an article described competencies for collaboration we asked ourselves the question: “is this something a doctor should know or do?” or, if not stated that clearly, “can we reasonably expect a doctor to influence the described condition, facilitator or barrier for collaboration?” If the answer was yes, this was seen as a competency or as a skill, knowledge or attitude.

### Critical appraisal

After a first screening of the selected articles it showed that most studies had a qualitative design, some had a quantitative or mixed method design or were opinion papers. We decided to use the questions proposed by Kuper et al. for the appraisal of qualitative research [16]. We chose to use these questions since we felt they were also applicable to other research designs than qualitative research. Table 2 shows the questions and the topics we reviewed in qualitative and quantitative designs.

**Table 2:** Critical appraisal questions

Question proposed by Kuper et al [16]	Qualitative (COREQ questions [17])	Quantitative
Was the sample used in the study appropriate to its research question?	Sampling, method of approach, sample size, description of sample	Sample size, power calculation
Were the data collected appropriately?	Setting of data collection, presence of non-participants, description of interview guide, repeat interviews, audio*/*visual recording, field notes, duration, data saturation, transcripts returned	Collection method, blinding, randomisation
Were the data analysed appropriately?	Methodological orientation of study, number of data coders, description of the coding tree, derivation of themes, software used, participant checking	Description of analysis methods, appropriate methods used
Can I transfer the results of this study to my own setting?	Clarity of described themes and subthemes, presentation of quotations, consistency between findings and data presented	Presentation of findings, consistency between findings and data presented
Does the study address potential ethical issues, including reflexivity?	Characteristics of interviewers and research team, ethical review when necessary	Ethical review when necessary
Overall: is what the researchers did clear?	Overall: is what the researchers did clear?	Overall: is what the researchers did clear?

This critical appraisal was done by two researchers independently (the first author (MJ) and JG, MS, NS or CF), using four answer options: yes, no, partly or not applicable. We did not exclude articles based on quality, because of the low number of relevant articles and the aim of our research: to give the readers insight into knowledge on collaborative competencies found in literature. We did find, however, that we needed to provide the reader with information on the quality of the reviewed articles. Therefore we assigned a critical appraisal score (CAS) to each article that could be fully scored. An article that could be fully scored and scored yes on all questions was given a score of 12 out of 12 (2 points for each question). Articles with a score beneath 7 out of 12 were rated as low quality, articles with a score ≥ 7 were rated as sufficient quality.

### Analysis

**Fig. 1:**
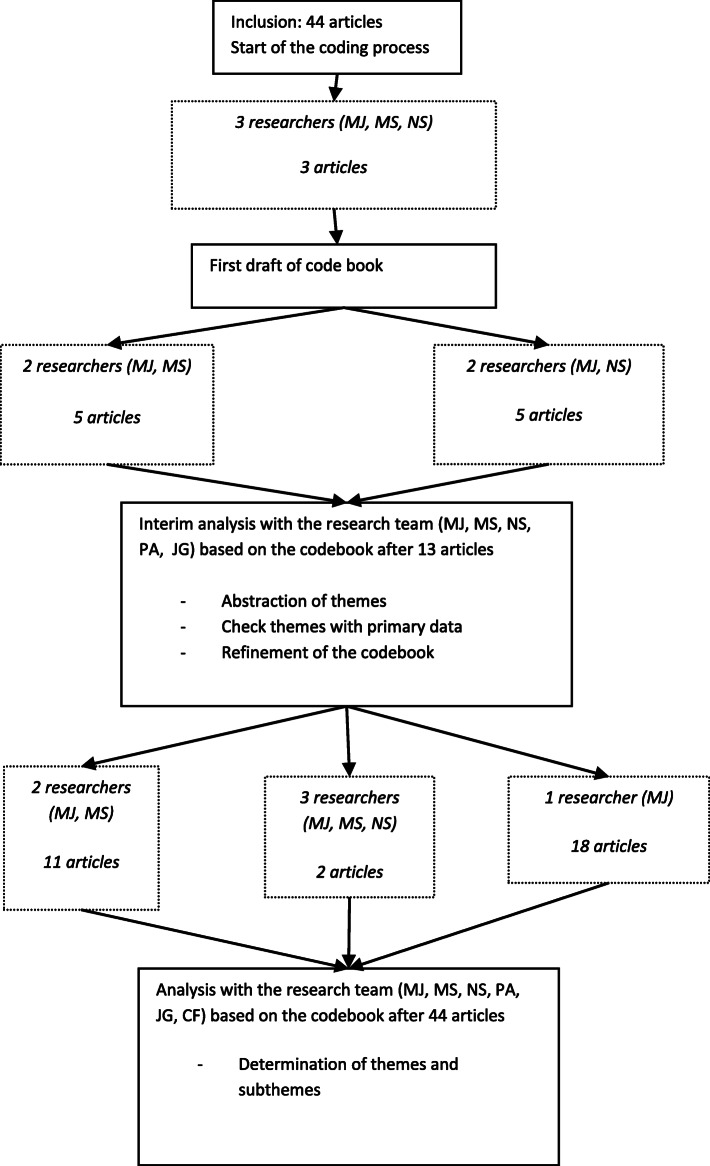
Coding process

We analysed the articles using thematic analysis to create a convergent qualitative synthesis [18, 19]. We followed the steps proposed in the article of Whittemore et al. concerning analysis of data in an integrative review: data reduction, data display, data comparison, conclusion drawing and data verification [12, 20]. We used an iterative approach, in which we moved back and forth between these steps. After getting acquainted with the material, we started coding relevant fragments (data reduction). We used open codes and coded only the results sections. We coded fragments as competencies when we judged that it described something a doctor should know or do. If it was not stated that clearly, for example in the case of a condition, facilitator or barrier for collaboration that could be influenced through knowledge or behaviour of a doctor, we also coded this as a competency. Three researchers with experience in qualitative research (MJ, MS, NS) were involved in the coding process. They coded the articles independently and met regularly to discuss their coding and solve discrepancies through discussion leading to the development of a codebook (data display) (Fig. 1). After coding 13 articles a discussion took place with the whole research team to identify the first themes (data comparison). After finishing the coding of all articles, the research team determined definitive themes and subthemes (conclusion drawing). Finally we compared themes with the primary data, the coded fragments (data verification) and checked how the contribution of articles without a CAS and low quality papers had influenced our conclusions. In the final three phases, we had several discussions within the research team before we were satisfied with the formulated themes. Atlas.ti7 version 7.1.5. was used to organise the data. The first author (MJ) kept a reflexivity journal to register decisions made during the analysis.

## Results

**Fig. 2:**
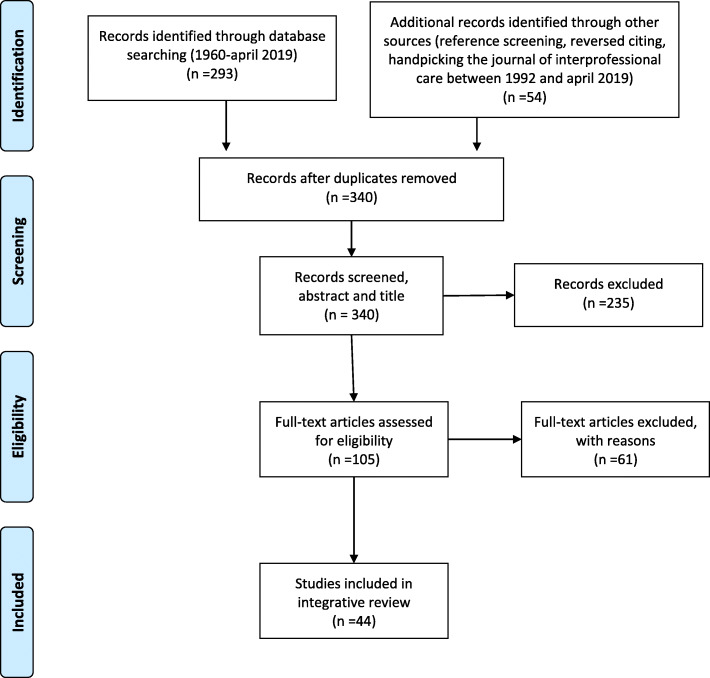
Inclusion process in Prisma Flow Diagram [65]

We included and coded 44 articles( [21–64] (Fig. 2 [65]). An overview of their characteristics is presented in Table S1 (additional file 2). Most articles used qualitative methods, several combined with quantitative research approaches, some used quantitative methods as surveys or questionnaires and a few articles were reviews or opinion papers. Six articles could not be fully scored due to the methods used in the article (opinion/discussion papers) [29, 32, 44, 45, 55, 61], so no CAS was assigned. Two articles scored ‘yes’ on all questions of the critical appraisal tool, resulting in a maximum CAS of 12 [27, 63]. Six articles were of low quality (CAS < 7 out of 12) [28, 41, 46, 49, 51, 58]. Most articles failed to fully address ethical issues, mainly reflexivity. Several articles did not discuss data saturation or did not state clearly which method of analysis was used (see additional file 3).

The various ways in which the papers described primary care doctors (PCDs) and secondary care doctors (SCDs) are displayed in Table 3. In the following parts of the article we use the abbreviations PCD and SCD. In the quotations we use the descriptions from the original articles. The quotations are mainly interpretations from the authors of the reviewed articles. One quotation is from a participant in the original research.

**Table 3:** Various types of primary and secondary care doctors as described in the retrieved articles

Primary care doctors (PCDs)	Secondary care doctors (SCDs)
General practitioners	Specialists
General health providers	Hospitalists
Generalists	Subspecialists
Family practitioners	Consultants
Primary care physicians	Specialist team
Referrers	
Requesters	
Callers	

Hardly any articles primarily addressed the collaborative competencies. Therefore, we had to derive competencies from experiences, motives, barriers and facilitators, or from conditions mentioned as necessary for collaboration between PCDs and SCDs. As explained in our methods section the following question was leading: “can we reasonably expect a doctor to influence the described condition, facilitator or barrier for collaboration?” Examples are the following fragments:

*“GPs feel undermined when some mental health providers refer their patients with physical health problems to other medical specialists without consulting the GP.” (van Hasselt et al.* [40]*)*

*“Family physicians, conversely, were annoyed when specialists "took over" their patients–particularly when they referred them to other specialists” (Beaulieu et al.* [19]*)*

Analysing these fragments, we judged that specialists could influence these situations through a change in behaviour and consequently coded these fragments as: “SCD discusses re-referral with the PCD”.

Overall, we identified six main themes within the collaborative competency domain: ‘patient-centred care: a common concern’, ‘collaborative attitude and respect’, ‘roles and responsibilities’, ‘mutual knowledge and understanding’, ‘communication’ and ‘leadership’. An overview of the themes and subthemes (i.e. associated knowledge, skills and attitudes) is given in Table 4.

**Table 4:** Themes and subthemes

**Theme**	**Subthemes (associated knowledge, skills and attitudes)**
**Patient-centred care: a common concern** ***Being able to work together with the same patient-centred goals***	Work from a common patient centred framework
	Respect and understand the patients’ view
	Know the background of the patient
	Set shared goals
	Shared care thinking
**Collaborative attitude and respect** ***Being able and willing to work together with respect for partners in collaboration***	Respect each other
	Respect each other’s roles, expertise and task distribution. Do not re-refer, do not retain
	Respect each other’s values related to the patients’ outcome
	Be willing to cooperate, be open-minded
	Look beyond one’s own position and task
	Accept limitations to autonomy
**Roles and responsibilities** ***Being able to know, make arrangements about, work in and follow up on a clear division of tasks, roles and responsibilities***	Understand, make arrangements about and cooperate in a clear division of tasks, roles and responsibilities
	Share responsibility for (continuing) care
	Give and receive feedback and address conflicts
	Be (directly) accessible and available
	Use consultation as an educational opportunity
	° Recognise moment, learning needs and sufficiency moment
	° Use and provide appropriate means
	° Deliver valuable information in a tactful way
	Specific responsibilities for PCD
	° Provide appropriate, timely referral
	° Follow up advice
	° Prepare the patients for the hospital visit
	Specific responsibilities SCD
	° Have clear referral requirements
	° Provide consultation
	° Include the PCD in important decision-making in the hospital
	° Guarantee that appropriate follow up is arranged
	° Confirm appropriate treatment plan, matched to the patient
	° Display interest in the patient and look for additional pertinent information
	° Take into account the referrer’s characteristics
	° Invite requesters for informal consultation
**Mutual knowledge and understanding** ***Being able to identify, know about and understand partners in collaboration***	Know your own limitations
	Know each other, doctors, personally
	Know the training, workplace and resources of the other
	Know the referral and communication system
	Understand the profession and perspective of the other and be able to match expectations
	Understand team dynamics and power relations
**Communication** ***Being able to communicate well in the right way on the right moment***	What should be communicated
	° Physical symptoms and medication: new, dose, duration, changes
	° Communicate only appropriate and essential information
	° Communicate the ending of consultation
	° Good request: states the urgency, a clear question and includes all the necessary information, including psychological information when relevant
	° Good reply: addresses the question asked, is prompt, adequate, detailed, shows understanding of the patient, includes prognosis, a treatment plan, future steps and follow up
	When should communication take place
	° Timely: sometimes this means quick, other times this means knowing when to communicate based on the medical situation
	° Immediate and direct in emergency situations
	° At admission, at major decision-making points and at discharge
	How should communication take place
	° Way of communication depends on urgency request
	° Communicate concise
	° Communicate without condescension
	Good oral communication
	° Communicate friendly, clearly (tone, pace, accent) and listen open and active
	° Ensure that it’s heard and understood
	° Communicate diplomatic with patients about colleagues
	Good written communication
	° Well-structured written communication by PCD: Provide referral reason and clinical information in the referral letter
	° Well-structured written communication by SCD: Summarise findings, assessment and management plan in a comprehensive non-discourteous letter, with bulleted recommendations at the bottom of response
**Leadership** ***Being able to show leadership to facilitate collaboration***	Manage persons (mediate, motivate, influence, build teams and relationships)
	Manage processes to facilitate collaboration (coordinate, organise well-functioning systems for collaboration, plan collaboration processes)
	Show leadership on organisational level and beyond to support collaborative practice

### Theme 1: patient-centred care: a common concern

Several articles [24, 45, 49, 55, 60](2 articles with no CAS, 1 article with a low CAS) emphasised the importance of patient-centredness and mutuality by describing collaboration between PCDs and SCDs as patient-centred, based on setting shared goals and working with a common aim. This is illustrated by the following fragment from a study investigating what motivates SCDs to initiate and sustain new models for collaboration with PCDs: *“The specialists felt that patients should be cared for by a qualified team, possibly with the GP in a central role. For this to work effectively, however, everybody involved has to have a good understanding of the common goals.” (Berendsen* et al. [24]*)*

### Theme 2: collaborative attitude and respect

Many articles [22, 23, 28, 30, 31, 33, 34, 36, 38, 40, 43, 45–47, 49, 53, 58, 60, 64] (4 low quality articles and no articles with no CAS) described that collaboration asks for a certain attitude of all participating doctors, as expressed in the following statement: *“Better cooperation is likely to be accompanied by a change in attitude from “mine” and “your” patients or tasks to “ours”” (Kvamme* et al. [45]*)*

Participants in collaboration should respect each other and see each other as equals. They should be open minded and willing to look beyond one’s own position. The following fragment further illustrates this theme: *“I [Primary care physician] would like [to get] the sense more of teamwork….I would like to be thought of and operate as a respected colleague who actually probably knows the patient much, much better and will see the patient quite frequently……” (Greer* et al. [38]*)*

In the review of Dossett et al. PCDs expressed their desire to be involved when patients are undergoing active treatment in the hospital, but in practice often felt excluded [30].

### Theme 3: roles and responsibilities

Another recurrent theme was ‘roles and responsibilities’ [21–31, 33–38, 40–43, 45, 47–49, 51–58, 60, 61, 63, 64], including 4 articles with no CAS and 5 of low quality. Many articles [21–23, 27, 30, 34, 38, 41, 42, 49, 54, 55, 58, 60, 63] indicated the importance of that roles and responsibilities should be clear, known and acted upon by all participants in collaboration. PCDs and SCDs differ in their roles and associated responsibilities as referrer and consultant. These specific responsibilities can be found in Table 3.

One study showed that ambiguous roles and responsibilities hindered good referrals: *“Participants reported a clear disagreement over which provider (subspecialist vs. primary care physician) was responsible for specific tasks during various parts of the referral process, including information gathering, patient workup, and follow-up.” (Hysong* et al. [42]*)*

Two articles focused on the learning-teaching interaction that can occur in consultations [48, 57]. According to these articles engaging in this interaction is a responsibility doctors should take. However, in a qualitative study of Marshall this interaction was also seen as a way to acquire other collaborative competencies: *“Education should be a two-way process since this will help promote mutual understanding of different roles and functions within the medical profession.” (Marshall* [48]*).*

### Theme 4: mutual knowledge and understanding

Several articles [22–24, 26, 29, 37, 40, 42, 43, 45–47, 49, 50, 53, 56, 60, 61, 63](2 with a low CAS and 3 with no CAS) mentioned the importance of knowledge about the partners in collaboration, their contexts and how this influences the way they work.

Both partners in the collaborative process asked for understanding, as seen in the following fragments: *“Specialists criticized GPs for not understanding the stress under which they now worked in hospitals” (Marshall* [47]*)*

*“All the interviewees considered it important for the specialists to increase and improve their understanding of the GPs' working method and the competencies associated with the profession of family medicine.” (Berendsen et al.* [23]*)*

In their study Wadhwa et al. described the tensions in interdoctor telephone consultations. One source of tension is the differing context, asking for understanding of the profession and the perspective of the other. *“The differing contexts in which the callers and consultants work were repeatedly described as a source of tension. When placing a telephone consultation, the caller is working from a context in which he or she is dealing with a difficult case outwith his or her level of expertise, creating an increased level of urgency from caller’s point of view. In contrast, however, for the consultants, answering telephone consults is 1 of many daily activities and is often not viewed as a high priority.” (Wadhwa* et al. [63]*)*

### Theme 5: communication

Almost all articles, including 6 with no CAS and 6 with a low CAS, emphasised the importance of good communication [21, 22, 25–29, 31, 32, 34, 36, 38–47, 49, 51–56, 58–64].

In a qualitative study of GP-led integrated diabetes care in primary health care it was stated that *“good communication and information sharing about patient care was core work” (Foster* et al. [36]*)*.

Studies described how, when and what should be communicated. A few articles focused solely on written communication with recommendations for referral and for reply letters [39, 59, 62, 64]. More detailed results can be found in Table 4.

### Theme 6: leadership

The importance of leadership in collaborative practice was discussed in a number of articles [36, 45, 51, 58, 60, 61] of which 2 with no CAS and 2 with a low CAS. Leadership could be demonstrated at three levels: 1. in relationships with other persons (e.g. mediate, motivate, influence), 2. in the ability to manage processes to facilitate collaboration. For example Sibert et al. described that *“The consultant must improve administrative and secretarial efficiency in order to prevent subsequent problems in communication or delay in appropriate care.” (Sibert* et al. [58]) and 3. in showing leadership at a system level to create an environment in which primary-secondary care collaboration is promoted and facilitated. This is presented in a statement from the European Working Party on Quality in Family Practice: *“For all healthcare systems the development of leadership is an important target for quality improvement at the primary/secondary care interface. This should involve regional administrators, local authorities, and healthcare professionals. Leaders need both to build effective teams and to delegate power and responsibilities if they are to promote really effective quality development.” (Kvamme* et al. [45]*)*

## Discussion

This review provides an integrative view on what competencies doctors need to provide good collaboration at the primary-secondary care interface. These competencies can be grouped into six themes that reflect different aspects of this collaboration: ‘patient-centred care: a common concern’, ‘collaborative attitude and respect’, ‘roles and responsibilities’, ‘mutual knowledge and understanding’, ‘communication’ and ‘leadership’. This variety shows the complexity of primary-secondary care collaboration.

Patient-centred care, the first theme, is nowadays a key element of high-quality care in many countries. From that scope collaboration is vital [66]. It is known from literature about teamwork that, in order to collaborate, team members should have a clear common purpose [67].

Good collaborative practice asks for a collaborative, respectful attitude from PCDs and SCDs, our second theme. In hierarchal practices like medicine, collaboration can be influenced by power relations [68]. It is described that lack of respect from SCDs hindered PCDs in their collaborative practice [69]. An underlying cause may be the different paradigms that reign in primary and secondary care respectively [70]. In our review participants from both primary and secondary care emphasised the importance of a collaborative, respectful attitude.

Unclear roles and responsibilities were often named as barriers for good collaboration. This is also found in other research about collaboration. In a review by Supper et al., determining facilitators and barriers for interprofessional (i.e. between different professionals) collaboration in primary care, the role of each professional was important: a lack of definition, awareness and recognition of these roles appeared to be a barrier for collaboration [71].

The theme of roles and responsibilities has a close relation with our fourth theme, mutual knowledge and understanding. Although it is impossible to know all collaborative partners personally, it is possible and needed to have an idea about the context they are working in. Strategies that can help improve this knowledge are traineeships in the work contexts of collaboration partners, observation of each other’s work in practice, directly asking the other about his or her context or actively sharing information about your context with your collaboration partner. This last strategy is part of general practice programmes that emphasise the importance of promoting the expertise and role of general practice to SCDs [69, 72].

The fifth theme, communication, is an essential aspect of collaboration. This theme is intertwined with the other themes. Good communication should take place in a respectful way and is largely based on knowledge about what the other needs to continue caring for the patient.

Our last theme, leadership, focuses on the power of doctors to change the practice they are working in, to facilitate collaboration. Similarities can be seen with the leader role of the CanMEDS model where one of the key competencies is described as: “contribute to the improvement of the health care delivery teams, organisations and systems” [7].

This review includes publications from 1990 till 2019, a period in which health care and the primary-secondary care interface have changed. However, the themes and subthemes concerning collaborative competencies are found diffusely over the publications suggesting that they are not very sensitive to time or a changing context.

### Implications for practice

To provide high-standard patient-centred care good collaboration is vital. Collaboration is complex and challenges lie both at personal and at organisational levels. These levels are related with each other [73]. The identified competencies in this review show that doctors play an important role in collaborative practice, not only in the way they act themselves but also in how they influence the organisational level.

The knowledge gathered in this review gives insight into what doctors need to know and show to realise good collaboration at the primary-secondary care interface. To improve collaborative behaviour, the first step is to know what desired behaviour contains. The results from this review can be used as a framework to get insight into deficiencies in collaborative behaviour and for (self)evaluation purposes. Furthermore, the knowledge can be used for formulating learning goals in graduate, post-graduate and continuing medical education.

Improving and learning collaboration in practice is a challenge, as workload is high and several tasks compete for the doctor’s attention. However, it is especially in this daily practice that most of the doctors’ competencies are acquired through workplace learning [74, 75]. Formulating more concrete learning goals for primary-secondary care collaboration can help to learn and improve collaboration in daily practice. The ability to learn from this daily practice could be enhanced through interaction, such as joint reflection and feedback [76, 77].

### Strengths & Limitations

To our knowledge this is the first review on collaborative competencies for doctors require to work optimally at the primary-secondary care interface. We used collaboration as a broad term and did not limit in patient groups or specialty. We used a rigorous method allowing inclusion of various types of literature.

Although we used various and broad search terms, during our analysis we found several other terms referring to the collaboration between PCD and SCD for example, relationship, consultation and referral. Therefore, we may have missed articles. However, we do not expect the themes to change with the inclusion of more articles, since no new themes emerged after the analyses of the first 13 articles of our set of 44 articles, indicating saturation of the information.

Another limitation is that we chose to include all papers to capture the full depth and breadth of the topic. We realise that the opinion papers were not peer reviewed and that quality is difficult to determine. Furthermore, six research articles were of low quality according to the critical appraisal score. This could affect the rigour of our findings. Therefore, we provided information on quality scores in the results section, showing the quality of the underlying papers which contributed to the themes. Especially the themes of ‘Leadership’ and ‘Patient-centered care: a common concern’ are based on few articles of which about half are opinion and low quality research articles. Based on discussions within the team we judged that the opinion papers (for example the article of Kvamme et al. [45] that presents recommendations from a European working group for working at the primary secondary care interface) did function as valuable sources to inform practice. Others, however, may judge differently. In qualitative analysis a theme can be based on little data. The iterative process and composition of the research team were important to create rigour and credibility of the data. Research discussions in our team, composed of researchers from different backgrounds, played an important role in formulating themes. However, the combination of little data and low quality data can affect the credibility of the data. Furthermore, all researchers work and/or teach at the primary-secondary care interface and their experiences may have influenced the analysis.

Not many articles discussed the competencies necessary for good collaboration directly. As a consequence, we had to derive competencies, which may have decreased the credibility of the data. To be sure that the derived competencies represent those necessary in practice, the competencies should be checked in practice by asking the question directly to stakeholders in collaboration, including the patients. It is remarkable that patients were hardly ever included in the research papers.

Last, we realise that most of the included studies were executed in developed countries, this could affect the transferability of our findings.

## Conclusion

This review provides an integrative view on competencies necessary for collaborative practice at the primary-secondary care interface. They are part of several domains, showing the complexity of collaboration. Doctors play an important role, not only in the way they collaborate in individual patient care, but also in how they help shaping organisational preconditions for collaboration. Acquirement of these competencies mainly takes place in the doctor’s workplace. The information gathered in this review can support doctors to further enhance and learn the various aspects of collaboration in daily practice and can be applied in educational programs during graduate, postgraduate and continuing medical education, thereby serving the final goal of improving patient care.

## Supplementary information

**Additional file 1.** Full list of search terms.

**Additional file 2: Table S1.** Characteristics of included articles (because of width as additional file).

**Additional file 3.** Critical appraisal included articles.

**Additional file 4.** Enhancing transparency in reporting the synthesis of qualitative research: the ENTREQ statement.

## Data Availability

All included literature can be found in the reference list and in Table S1 (additional file 2). The datasets (Atlas.ti files) used during the study are available from the corresponding author on reasonable request.

## Abbreviations

GP: General practitioner
PCD: Primary care doctor
SCD: Secondary care doctor
