# Supplementary material for: Competencies to promote collaboration between primary and secondary care doctors: an integrative review
Source: BMC Fam Pract. 2020 Sep 2;21:179. doi: 10.1186/s12875-020-01234-6 (PMC7469099; doi:10.1186/s12875-020-01234-6)
Supplement: Supplementary file 1 — Additional file 1. Full list of search terms. [file 12875_2020_1234_MOESM1_ESM.docx]

**Additional file 1: Search terms per database**

**Research question**

“Which competencies do doctors need to promote collaboration in order to provide good patient care at the primary-secondary care interface?”

Limit: language English

**Medline:**

1. **Interprofessional collaboration:**

***MESH-terms:***

- interprofessional relations
- interdisciplinary communication

**Free text:**

((((interprofessi* or interdisciplin* or multiprofessi* or transdisciplin* or multidisciplin* or cross) adj3 disciplinary) or cross?disciplin*) adj3 (communicat* or relat* or collabora* or cooperat*)).tw. (159)

((((interprofessi* or interdisciplin* or multiprofessi* or transdisciplin* or multidisciplin* or cross) adj3 disciplinary) or cross?disciplin*) adj3 (communicat* or relat* or collabora* or cooperat*)).kf. (1)

1. **Primary care**

***MESH terms:***

- Primary Health Care
- General Practitioners
- Doctor, family
- Doctor, primary care

***Free text:***

((primary adj3 care) or (healthcare adj3 primary)).tw.

((primary adj3 care) or (healthcare adj3 primary)).kf.

((general* adj1 practi*) or (family adj1 doctor*) or (general* adj3 practi* adj3 doctor*) or (primary adj3 care adj3 doctor*)).tw

((general* adj1 practi*) or (family adj1 doctor*) or (general* adj3 practi* adj3 doctor*) or primary adj3 care adj3 doctor*)).kf

1. **Secondary care**

***MESH terms:***

- medicine
- dermatology
- hospital medicine
- exp (explode) internal medicine
- neurology
- exp pediatrics
- exp psychiatry
- exp specialties, surgical

***Free text:***

((medical* adj3 special*) or (medic* adj3 internal) or (surg* adj3 special*) or (Accident adj1 emergen* adj1 medicin*) or Allergolog* or Anaesthe* or hematolo* or cardiolog* or psychiat* or surge* or Dermatolog* or Endocrinolog* or Gastroenterolog* or Geriat* or Immunolog* or Infecti* or Intern* or Microbiolog* or Nephrol* or Neurolog* or Nuclear medicin* or Obstetric* or Gynecolog* or Ophthalmolog* or Orthopaedic* or Otorhinolaryngolog* or Paediatric* or Patholog* or Pharmacolog* or (Physic* adj1 medicin adj3 rehabilitation) or Radiolog* or Radiotherap* or (Respirator* adj1 medicin*) or oncolog* or pulmon* or Rheumatolog* or Urolog*).tw.

((medical* adj3 special*) or (medic* adj3 internal) or (surg* adj3 special*) or (Accident adj1 emergen* adj1 medicin*) or Allergolog* or Anaesthe* or hematolo* or cardiolog* or psychiat* or surge* or Dermatolog* or Endocrinolog* or Gastroenterolog* or Geriat* or Immunolog* or Infecti* or Intern* or Microbiolog* or Nephrol* or Neurolog* or Nuclear medicin* or Obstetric* or Gynecolog* or Ophthalmolog* or Orthopaedic* or Otorhinolaryngolog* or Paediatric* or Patholog* or Pharmacolog* or (Physic* adj1 medicin adj3 rehabilitation) or Radiolog* or Radiotherap* or (Respirator* adj1 medicin*) or oncolog* or pulmon* or Rheumatolog* or Urolog*).kf.

1. **Competencies:**

***MESH terms:***

- Health Knowledge, Attitudes, Practice
- Clinical competence
- Professional competence

***Free text:***

((competenc*) or (clinic* adj3 competenc*) or (clinic* adj3 skill*) or ((knowledge) or (attitudes) or (practice) adj3 (health))).tw

((competenc*) or (clinic* adj3 competenc*) or (clinic* adj3 skill*) or ((knowledge) or (attitudes) or (practice) adj3 (health))).kf

**CINAHL:**

**Search terms**

((interprofessional relations) or (collaboration)) and ((primary care doctor) or (general practitioner) or (family doctor)) and ((secondary care) or (hospital))

**Psychinfo:**

**Search terms**

1. **Interprofessional collaboration**

***Subheadings***

- collaboration/ or
- interpersonal interaction/

***Free text:***

|  | ((((interprofessi* or interdisciplin* or multiprofessi* or transdisciplin* or multidisciplin* or cross) adj3 disciplinary) or cross?disciplin*) adj3 (communicat* or relat* or collabora* or cooperat*)).tw. |
| --- | --- |
|  | ((((interprofessi* or interdisciplin* or multiprofessi* or transdisciplin* or multidisciplin* or cross) adj3 disciplinary) or cross?disciplin*) adj3 (communicat* or relat* or collabora* or cooperat*)).id |

1. **Primary care**

***Subheadings***

- Primary Health Care
- General Practitioners
- Doctor, family
- Family medicine

***Free text***

((primary adj3 care) or (healthcare adj3 primary)).tw.

((primary adj3 care) or (healthcare adj3 primary)).id

((general* adj1 practi*) or (family adj1 doctor*) or (general* adj3 practi* adj3 doctor*) or (primary adj3 care adj3 doctor*)).tw

((general* adj1 practi*) or (family adj1 doctor*) or (general* adj3 practi* adj3 doctor*) or primary adj3 care adj3 doctor*)).id

1. **Secondary care**

***Subheadings***

doctors/ or gynecologists/ or internists/ or neurologists/ or obstetricians/ or pathologists/ or pediatricians/ or psychiatrists/ or surgeons/ or clinicians/

***Free text***

((medical* adj3 special*) or (medic* adj3 internal) or (surg* adj3 special*) or (Accident adj1 emergen* adj1 medicin*) or Allergolog* or Anaesthe* or hematolo* or cardiolog* or psychiat* or surge* or Dermatolog* or Endocrinolog* or Gastroenterolog* or Geriat* or Immunolog* or Infecti* or Intern* or Microbiolog* or Nephrol* or Neurolog* or Nuclear medicin* or Obstetric* or Gynecolog* or Ophthalmolog* or Orthopaedic* or Otorhinolaryngolog* or Paediatric* or Patholog* or Pharmacolog* or (Physic* adj1 medicin adj3 rehabilitation) or Radiolog* or Radiotherap* or (Respirator* adj1 medicin*) or oncolog* or pulmon* or Rheumatolog* or Urolog*).tw.

((medical* adj3 special*) or (medic* adj3 internal) or (surg* adj3 special*) or (Accident adj1 emergen* adj1 medicin*) or Allergolog* or Anaesthe* or hematolo* or cardiolog* or psychiat* or surge* or Dermatolog* or Endocrinolog* or Gastroenterolog* or Geriat* or Immunolog* or Infecti* or Intern* or Microbiolog* or Nephrol* or Neurolog* or Nuclear medicin* or Obstetric* or Gynecolog* or Ophthalmolog* or Orthopaedic* or Otorhinolaryngolog* or Paediatric* or Patholog* or Pharmacolog* or (Physic* adj1 medicin adj3 rehabilitation) or Radiolog* or Radiotherap* or (Respirator* adj1 medicin*) or oncolog* or pulmon* or Rheumatolog* or Urolog*).id.

1. **Competencies**

***Subheadings***

- Competence
- Professional competence
- Social skills
- health knowledge
- "knowledge (general)
- health attitudes
- health behavior

***Free text***

((competenc*) or (clinic* adj3 competenc*) or (clinic* adj3 skill*) or ((knowledge) or (attitudes) or (practice) adj3 (health))).tw

((competenc*) or (clinic* adj3 competenc*) or (clinic* adj3 skill*) or ((knowledge) or (attitudes) or (practice) adj3 (health))).id

**ERIC**

**Search terms**

1. **Interprofessional collaboration**

***Subheadings***

- interprofessional relationship/ or
- interpersonal relationship

***Free text:***

| ((((interprofessi* or interdisciplin* or multiprofessi* or transdisciplin* or multidisciplin* or cross) adj3 disciplinary) or cross?disciplin*) adj3 (communicat* or relat* or collabora* or cooperat*)).tw. |
| --- |
| ((((interprofessi* or interdisciplin* or multiprofessi* or transdisciplin* or multidisciplin* or cross) adj3 disciplinary) or cross?disciplin*) adj3 (communicat* or relat* or collabora* or cooperat*)).id |

1. ***Primary care***

***Subheadings***

- primary health care
- "family practice (medicine)

***Free text***

((primary adj3 care) or (healthcare adj3 primary)).tw.

((primary adj3 care) or (healthcare adj3 primary)).id

((general* adj1 practi*) or (family adj1 doctor*) or (general* adj3 practi* adj3 doctor*) or (primary adj3 care adj3 doctor*)).tw

((general* adj1 practi*) or (family adj1 doctor*) or (general* adj3 practi* adj3 doctor*) or (primary adj3 care adj3 doctor*)).id

1. ***Secondary care***

***Subheadings***

- medicine
- anesthesiology
- geriatrics
- gynecology
- internal medicine
- neurology
- obstetrics
- oncology
- ophthalmology
- pathology
- pediatrics
- psychiatry
- surgery
- toxicology

***Free text***

((medical* adj3 special*) or (medic* adj3 internal) or (surg* adj3 special*) or (Accident adj1 emergen* adj1 medicin*) or Allergolog* or Anaesthe* or hematolo* or cardiolog* or psychiat* or surge* or Dermatolog* or Endocrinolog* or Gastroenterolog* or Geriat* or Immunolog* or Infecti* or Intern* or Microbiolog* or Nephrol* or Neurolog* or Nuclear medicin* or Obstetric* or Gynecolog* or Ophthalmolog* or Orthopaedic* or Otorhinolaryngolog* or Paediatric* or Patholog* or Pharmacolog* or (Physic* adj1 medicin adj3 rehabilitation) or Radiolog* or Radiotherap* or (Respirator* adj1 medicin*) or oncolog* or pulmon* or Rheumatolog* or Urolog*).itw

((medical* adj3 special*) or (medic* adj3 internal) or (surg* adj3 special*) or (Accident adj1 emergen* adj1 medicin*) or Allergolog* or Anaesthe* or hematolo* or cardiolog* or psychiat* or surge* or Dermatolog* or Endocrinolog* or Gastroenterolog* or Geriat* or Immunolog* or Infecti* or Intern* or Microbiolog* or Nephrol* or Neurolog* or Nuclear medicin* or Obstetric* or Gynecolog* or Ophthalmolog* or Orthopaedic* or Otorhinolaryngolog* or Paediatric* or Patholog* or Pharmacolog* or (Physic* adj1 medicin adj3 rehabilitation) or Radiolog* or Radiotherap* or (Respirator* adj1 medicin*) or oncolog* or pulmon* or Rheumatolog* or Urolog*).id.

1. **Competencies**

***Subheadings***

- competence/ or
- interpersonal competence/ or
- skills/
- knowledge level/ or
- performance/

***Free text***

((competenc*) or (clinic* adj3 competenc*) or (clinic* adj3 skill*) or ((knowledge) or (attitudes) or (practice) adj3 (health))).tw

((competenc*) or (clinic* adj3 competenc*) or (clinic* adj3 skill*) or ((knowledge) or (attitudes) or (practice) adj3 (health))).id
