# Supplementary material for: Competencies to promote collaboration between primary and secondary care doctors: an integrative review
Source: BMC Fam Pract. 2020 Sep 2;21:179. doi: 10.1186/s12875-020-01234-6 (PMC7469099; doi:10.1186/s12875-020-01234-6)
Supplement: Supplementary file 2 — Additional file 2: Table S1. Characteristics of included articles (because of width as additional file). [file 12875_2020_1234_MOESM2_ESM.docx]

Table 3: Characteristics of included articles

| **Author** | **Aim** | **Health care setting/specific patient group** | **Method** | **Method of analysis** | **Participants** | **Country** | **Year** |
| --- | --- | --- | --- | --- | --- | --- | --- |
| Beaser(1) | To identify consultation and communication practices between PCDs and specialists in diabetes care and to ascertain the actual utilization of guidelines.   To understand educational needs among providers with respect to potential efforts to increase the efficacy and influence of current guidelines. | Diabetes care | Questionnaires | Descriptive statistics | 491 primary care providers (doctors, osteopathic doctors and nurses)   249 specialists (89% endocrinologists) | USA | 2011 |
| Beaulieu(2) | To explore how collaboration between family doctors and specialists is approached in the formal curriculum of such programs.  To explore how it is experienced day-to-day in academic medical centers that constitute for trainers and trainees as a workplace. | No specification | Interviews: individual and group interviews  Review of programs | Immersion/crystallization method | 4 deans  28 program directors/educators 47 residents  4 residency programs: family practice, psychiatry, internal medicine, radiology | Canada | 2009 |
| Berendsen(3) | To answer the questions What motivates GPs to initiate and sustain new models for collaborating with medical specialists?  What kinds of new models of collaborative practice do GPs suggest? | No specification | Semi-structured interviews | Framework method | 21 GPs | Netherlands | 2007 |
| Berendsen(4) | To explore what the medical specialists motivates to initiate and sustain new models for collaborating with GPs. | No specification | Semi-structured interviews | Framework method | 18 specialists (surgeons, radiologists, pediatricians, psychiatrists, doctors in internal medicine) | Netherlands | 2006 |
| Bethell(5) | To study general practitioners' and head and neck surgeons' experiences with a two-week wait referral system. | Head and neck surgery | Questionnaires | Unspecified | 27 GPs 15 head and neck surgeons | UK | 2015 |
| Boulware(6) | To provide current information on how consultations can be most effective and how EMR systems might improve consultations. | No specification | Survey | Descriptive and inferential statistics | 323 doctors (PCPs and specialists and their residents) of 4 teaching hospitals. | USA | 2009 |
| Chong(7) | To identify facilitators en barriers for interprofessional collaboration and shared decision making in mental health care according to healthcare providers. | Mental health care | In depth semi-structured interviews | Thematic analysis | 4 psychiatrists 4 GPs 11 pharmacists 7 mental health nurses 5 paramedic professionals (occupational therapists, psychologists, social workers) | Australia | 2013 |
| Desmedt(8) | To ascertain family doctors' opinions of the home care services dispended by a specialist palliative care team. | Palliative care | Questionnaires and mirror questionnaires | Descriptive statistics | 50 family doctors of 50 deceased patients   palliative team: 1 consultant palliative care, 4 nurses, 1 social worker, secretaries | Belgium | 2002 |
| Doohan(9) | To propose a solution in the form of a chief primary care officer to restore continuity ar the primary-secondary care interface | No specification | Reflection paper | na | na | USA | 2017 |
| Dossett(10) | To describe the attributes of the relationship and communication between PCPs and cancer specialists | Cancer care | Review | Thematic analysis | na | USA | 2017 |
| Eyers(11) | To find out how satisfied referrers were with the service (mood disorder unit).  To define the elements of referrer satisfaction.  To examine differences in referral priorities between GPs and psychiatrists.  To define the needs of referrers. | Mental health care | Survey | Descriptive and inferential statistics | referrers of the first 308 patients (75 Psychiatrists, 59 GPs, 13 Others) | Australia | 1994 |
| Farnan(12) | To illustrate the major domains of communication for hospitalists and the influence on the quality of care and to identify common barriers and strategies to overcome them. | Hospital care | Description of a case scenario | na | na | USA | 2006 |
| Fickel(13) | To provide a foundation for understanding current provider practices in collaboration in mental health care, examining their ideas towards barriers and identify strategies to overcome them. | Mental health care | Semi-structured interviews | Qualitative, unspecified | 10 primary care clinic leaders and 12 mental health clinic leaders (16 doctors, 5 nurses/nurse practitioners, 1 psychologist) | USA | 2007 |
| Firn(14) | To assess existing evidence of inpatient generalist palliative care providers' perceptions of what facilitates or hinders collaboration with hospital based teams. | Palliative care | Literature review | Narrative literature synthesis | 23 studies | USA | 2015 |
| Fleury(15) | To identify the clinical and collaborative practices of general practitioners in Quebec in managing patients with mental health problems. | Mental health care | Interviews  Survey | Qualitative, unspecified  Descriptive and inferential statistics | Interviews: 60 GPs  Questionnaire: 398 GPs | Canada | 2012 |
| Foster(16) | To explore the experiences of multidisciplinary team members with an GP-led integrated diabetes care innovation in primary care to inform a broader implementation strategy. | Diabetes care | Focus groups  Interviews | Thematic analysis | 2 focus groups: 1 with 3 and 1 with 5 PCPs  Interviews: 2 endocrinologists, 2 practice managers | Australia | 2016 |
| Fredheim(17) | To investigate strengths en weaknesses in today's collaboration between GPs en specialised mental health services and suggest improvements for this collaboration. | Mental health care | Focus groups | Systematic text condensation | 6 Focus groups: 2 with 5 GPs, 1 with 6 nurses from primary care, 2 with 3 nurses from secondary care and 1 with 6 residents in psychiatry | Norway | 2011 |
| Greer(18) | To identify modifiable patient, provider and system-level barriers they face to adequately preparing patients for renal replacement therapy that could be targeted for future interventions. | Nephrology | Interviews | Grounded theory | 6 nephrologists, 3 doctor assistants, 1 specialized nurse, 4 PCPs | USA | 2015 |
| Grol(19) | To determine to which extent specialist’ reply letters are related to the referral letters and if better referral letters produce better reply letters. | No specification | Assessment of the letters.  Referral letters by 2 assessors, reply letters by 3 assessors with quality criteria based on literature | Descriptive and inferential statistics | 637 referral letters and associated reply letters of 42 GP practices | Netherlands | 2003 |
| van Hasselt(20) | To develop advices for implementation to improve the physical health of severe mental illness patients. | Mental health care | Modified Delphi, 3 rounds | Thematic analysis Descriptive statistics | 10 patients, 13 family members, 5 GPs, 8 mental health care professionals | Netherlands | 2015 |
| Hayes(21) | To gain a better understanding of the issues and challenges between generalists and sleep specialists that might undermine optimal patient care for patients with sleep disorders focusing on obstructive sleep apnea (OSA) and shift work disorder (SWD). | Patients with sleep disorders | Focus groups   Interviews  Survey | Qualitative analysis, unspecified  Descriptive and inferential statistics | 5 focus groups about OSA: 20 generalists and 12 specialists  24 interviews about SWD: 16 generalists and 8 specialists  Survey: 145 generalists about OSA, 200 generalists and 100 specialists about SWD | USA | 2012 |
| Hysong(22) | To provide insight into the achievement of 3 integrating conditions (accountability, predictability en common understanding) in the referrals process by identifying barriers, facilitators and perceived solutions for improving communication and coordination of EHR-based referrals in an integrated healthcare system. | No specification | Interviews  Focus groups | Qualitative analysis, unspecified Techniques adapted from grounded theory and content analysis | Interviews: 7 key experts (subspecialists, doctor assistants, administrative supportive staff) in referral in subspecialties (cardiology, neurology, pulmonology, gastroenterology)  6 Focus groups: 3 with PCPs, 3 with subspecialists, a total of 30 doctors | USA | 2011 |
| Janssen(23) | To explore what and how trainees learned by participating in a consultation programme | Internal medicine | Focus groups | Qualitative content analysis | 3 Focus groups: 1 with GP-trainees, 1 with internal medicine-trainees, 1 with GP-and internal medicine-supervisors | Netherlands | 2017 |
| Kessler(24) | To explore existing models for improving provider-to-provider communication with a focus on consultation. Highlight the ER as a unique training environment for training in interprofessional communication skills. | No specification ER specially named | Literature review  Expert opinions | na | na | USA & Canada | 2013 |
| Kvamme(25) | To outline the recommendations of the European Working Party on Quality in Family Practice on how cooperation between general practitioners and specialists might be improved. | No specification | Recommendations from a work group | na | composition work group:12 primary care providers from Europe | Europe | 2001 |
| Langley(26) | To develop an empirical definition of exemplary clinical performance. | No specification | Interviews  Ranking of answers in order of importance | Description of answers, frequency and importance | 25 family doctors 25 specialists (doctors in internal medicine, psychiatrists, surgeons, gynaecologists, paediatricians) | Canada | 1989 |
| Marshall(27) | To investigate the professional relationships in terms of balance and conflict between GPs en specialist. | No specification | Interviews  Focus groups | Content analysis | Interviews: 12 GPs, 12 specialists  4 Focus groups: each with 4 GPs and 4 specialists | UK | 1998 |
| Marshall(28) | To identify barriers for effective educational interaction between GPs en specialists. | No specification | Interviews   Focus groups | Content analysis | Interviews: 12 GPs, 12 specialists  4 Focus groups: each with 4 GPs and 4 specialists | UK | 1998 |
| Mertala(29) | To express the co-operative orientation of professionals; what does it mean to know together, what are boundaries en possibilities. | No specification | Questionnaires -Open ended -Combined graphical two-dimensional | Grounded theory approach   Descriptive statistics | 44 GPs 34 specialists | Finland | 2009 |
| Newton(30) | To identify factors that influence the referral of GPs. | No specification | Interviews | Grounded theory approach | 15 GPs | UK | 1993 |
| Norris(31) | To investigate what skills are important to realise collaboration between professionals working in managed clinical networks and investigate the training needs. | Cancer care | Focus group  Questionnaires based on focus groups  Post questionnaire interviews | Not described | Focus group: 5 nurses, 2 pharmacists, 1 manager 1 allied health care professional  Questionnaires: 88 doctors, 57 nurses, 38 allied health care professionals, 16 other  Interviews: 10 persons, not clear who, at least 4 doctors | Scotland | 2005 |
| Otte(32) | To identify possible difficulties and barriers to effective collaboration at the end-of-life between hospitalists and GPs. | End-of-life care | Interviews | Qualitative content analysis | 23 GPs | Switzerland | 2016 |
| Perley(33) | To define what “curbside” consultation is, how and why it is used and what the consequences for the medical library are. | No specification | Literature review  Observation  Interviews | Qualitative analysis, unspecified | Observation:16 PCPs  Interviews:16 PCPs and 28 specialists the PCPs contacted for consultation | USA | 2006 |
| Pinelli(34) | To assess and advance the understanding of the challenges in the discharge process from the perspective of all involved stakeholders | No specification | Interviews  Focus groups | Qualitative analysis, inductive category development approach | Interviews: 39 providers, 7 patients  8 Focus groups: 3 with physicians (n=14), 2 with nurses (n=11) 1 with care coordinators/social workers (n=5) 1 with consulting physicians (n=5), 1 with physical/occupational therapists/pharmacists (n=6) | USA | 2017 |
| Politi(35) | To review challenges in providing interprofessional patient decision support.  To propose suggestions for providing this.  To consider promising strategies for teaching this. | Specialty care | Review and expert opinion | na | Unclear, not documented | USA, Netherlands, Canada, UK, Germany, Italy | 2011 |
| Sampson(36) | To explore the relational perspectives of GPs and hospital specialists | No specification | Interviews | Thematic analysis | 10 GPs and 12 hospital specialists | Scotland | 2016 |
| Shershneva(37) | To form a theoretical framework for understanding learning in generalist-specialist consultations. | Internal medicine | Semi-structured interviews | Grounded theory approach | 4 family doctors 6 generalists in internal medicine 9 subspecialists in internal medicine | USA | 2006 |
| Sibert(38) | To identify skills required in contacts with referring doctors for effective consultation and produce a consensual definition of these skills. | Urology | Literature review   Focus groups | Qualitative analysis, unspecified | Focus groups with consultants: urologists, residents urology.  Focus groups with referrers: 2 nephrologists, 1 doctor in internal medicine, 1 doctor in geriatrics, 1 gastroenterologist, 1 primary care physician, 6 GPs | France | 2002 |
| Stalhammar(39) | To examine how GPs and specialists assess the importance of various aspects of information regarding cancer treatment in the discharge letter and how they perceive their counterparts opinion of the same aspects. | Cancer care | Questionnaires | Descriptive and inferential statistics | 204 GPs 48 specialists (medical and surgical oncologists) | Sweden | 2009 |
| Sunderji(40) | To identify the core competencies required for integrative care practice in Canada necessary for all psychiatric postgraduate learners. | Mental health care | Interviews  Modified delphi method | Thematic analysis  Descriptive statistics | 9 psychiatrists  32 experts in integrated metal health care | Canada | 2016 |
| Swar(41) | To outline common challenges and strategies based on a two-year study using a collaborative care model with teleconsultation to deliver care for depression, psychosis and posttraumatic stress disorder | Mental health care | Description  Expert opinion | na | na | Nepal | 2019 |
| Vargas(42) | To determine the level and characteristics of primary care and secondary care doctors’ use of referral and reply letters and to explore influencing factors | No specification | questionnaires | Descriptive and inferential statistics | 2160 primary and secondary care doctors | Argentina, Brazil, Chile, Colombia, Mexico, Uruguay | 2018 |
| Wadhwa(43) | To study the interdoctor telephone consultations in order to inform future communication skills initiatives in this domain. With a final goal to formally address them in medical training. | Paediatric infectious diseases | Documentation   Field observation  Semi-structured interviews | Description   Grounded theory approach | 129 telephone calls documented  51 hours observation of consultants (paediatric infectious disease service)  Interviews with 12 callers (community paediatricians en family practitioners) and 12 consultants | Canada | 2006 |
| Westerman(44) | To assess the quality of communication in letters between GPs and specialists. | No specification | Assessment of letters by judges (4 GPs and 4 specialists) based on previous set criteria | Descriptive and inferential statistics | 144 referrals: letters from and to the GP 1152 assessments | Netherlands | 1990 |
|  | PCP= primary care physician |  |  | na= not applicable | |  |  |
|  | GP= general practitioner |  |  | UK= United Kingdom | |  |  |
|  | EMR= electronic medical record |  |  | USA= United States of America | |  |  |
|  | EHR= electronic health records |  |  | Year = publication year |  |  |  |
|  | ER= emergency room |  |  |  |  |  |  |

1. Beaser RS, Okeke E, Neighbours J, Brown J, Ronk K, Wolyniec WW. Coordinated primary and specialty care for type 2 diabetes mellitus, guidelines, and systems: an educational needs assessment. Endocrine Practice. 2011;17(6):880-90.

2. Beaulieu MD, Samson L, Rocher G, Rioux M, Boucher L, Del Grande C. Investigating the barriers to teaching family physicians' and specialists' collaboration in the training environment: a qualitative study. BMC Medical Education. 2009;9:31.

3. Berendsen AJ, Benneker WH, Meyboom-de Jong B, Klazinga NS, Schuling J. Motives and preferences of general practitioners for new collaboration models with medical specialists: a qualitative study. BMC Health Services Research. 2007;7:4.

4. Berendsen AJ, Benneker WH, Schuling J, Rijkers-Koorn N, Slaets JP, Meyboom-de Jong B. Collaboration with general practitioners: preferences of medical specialists--a qualitative study. BMC Health Serv Res. 2006;6:155.

5. Bethell G, & Leftwick, P. . Views of general practitioners and head and neck surgeons on the referral system for suspected cancer: A survey. The Journal of Laryngology & Otology. 2015;129(9):893-7.

6. Boulware DR, Dekarske AS, Filice GA. Physician preferences for elements of effective consultations. Journal of general internal medicine. 2010;25(1):25-30.

7. Chong WW, Aslani P, Chen TF. Shared decision-making and interprofessional collaboration in mental healthcare: a qualitative study exploring perceptions of barriers and facilitators. Journal of Interprofessional Care. 2013;27(5):373-9.

8. Desmedt M, Michel H. Palliative home care: improving co-operation between the specialist team and the family doctor. Supportive Care in Cancer. 2002;10(4):343-8.

9. Doohan N, DeVoe J. The Chief Primary Care Medical Officer: Restoring Continuity. Ann Fam Med. 2017;15(4):366-71.

10. Dossett LA, Hudson JN, Morris AM, Lee MC, Roetzheim RG, Fetters MD, et al. The primary care provider (PCP)-cancer specialist relationship: A systematic review and mixed-methods meta-synthesis. CA: a cancer journal for clinicians. 2017;67(2):156-69.

11. Eyers K, Brodaty H, Roy K. What the doctor ordered? Referrer satisfaction with a mood disorders unit. Australian & New Zealand Journal of Psychiatry. 1994;28(3):498-504.

12. Farnan JJ, JK; Arora, V. Effective communication in the inpatient care setting: an essential competency for hospitalists. Hospital Physician. 2006;42(6):8.

13. Fickel JJ, Parker LE, Yano EM, Kirchner JE. Primary care - mental health collaboration: an example of assessing usual practice and potential barriers. J Interprof Care. 2007;21(2):207-16.

14. Firn J, Preston N, Walshe C. What are the views of hospital-based generalist palliative care professionals on what facilitates or hinders collaboration with in-patient specialist palliative care teams? A systematically constructed narrative synthesis. Palliative medicine. 2016;30(3):240-56.

15. Fleury MJ, Farand L, Aube D, Imboua A. Management of mental health problems by general practitioners in Quebec. Canadian Family Physician. 2007;58(12):e732-8, e25-31.

16. Foster M, Burridge L, Donald M, Zhang J, Jackson C. The work of local healthcare innovation: a qualitative study of GP-led integrated diabetes care in primary health care. BMC Health Serv Res. 2016;16:11.

17. Fredheim T, Danbolt LJ, Haavet OR, Kjonsberg K, Lien L. Collaboration between general practitioners and mental helath care porfessionals: a qualitative study. International Journal of Mental Health Systems. 2011;5(13).

18. Greer RC, Ameling JM, Cavanaugh KL, Jaar BG, Grubbs V, Andrews CE, et al. Specialist and primary care physicians' views on barriers to adequate preparation of patients for renal replacement therapy: a qualitative study. BMC nephrology. 2015;16:37.

19. Grol R, Rooijackers-Lemmers N, van Kaathoven L, Wollersheim H, Mokkink H. Communication at the interface: do better referral letters produce better consultant replies? British Journal of General Practice. 2003;53(488):217-9.

20. van Hasselt FM, Oud MJ, Loonen AJ. Practical recommendations for improvement of the physical health care of patients with severe mental illness. Acta Psychiatrica Scandinavica. 2015;131(5):387-96.

21. Hayes SM, Murray S, Castriotta RJ, Landrigan CP, Malhotra A. (Mis) perceptions and interactions of sleep specialists and generalists: obstacles to referrals to sleep specialists and the multidisciplinary team management of sleep disorders. Journal of Clinical Sleep Medicine. 2012;8(6):633-42.

22. Hysong SJ, Esquivel A, Sittig DF, Paul LA, Espadas D, Singh S, et al. Towards successful coordination of electronic health record based-referrals: a qualitative analysis. Implementation science : IS. 2011;6:84.

23. Janssen M, Sagasser MH, Laro EAM, de Graaf J, Scherpbier-de Haan ND. Learning intraprofessional collaboration by participating in a consultation programme: what and how did primary and secondary care trainees learn? BMC Med Educ. 2017;17(1):125.

24. Kessler CS, Chan T, Loeb JM, Malka ST. I'm clear, you're clear, we're all clear: improving consultation communication skills in undergraduate medical education. Academic medicine : journal of the Association of American Medical Colleges. 2013;88(6):753-8.

25. Kvamme OJ, Olesen F, Samuelsson M. Improving the interface between primary and secondary care: a statement from the European Working Party on Qualitu in Family Practice (EQuiP). Quality in Health Care. 2001;10:33-9.

26. Langley GR, Till JE. Exemplary family physicians and consultants: empirical definition of contemporary medical practice. CMAJ Canadian Medical Association Journal. 1989;141(4):301-7.

27. Marshall MN. How well do general practitioners and hospital consultants work together? A qualitative study of cooperation and conflict within the medical profession. British Journal of General Practice. 1998;48:1379-82.

28. Marshall MN. Qualitative study of education interaction between general practitioners and specialists. British Medical Journal. 1998;316:442-5.

29. Mertala S. How to know together? Physicians' co-orientation between hospitals and health centres. J Interprof Care. 2009;23(2):185-94.

30. Newton J, Hayes V, Hutchinson A. Collaboration between doctors: A study of referral decision making. J Interprof Care. 1993;7(3):269-79.

31. Norris E, Alexander H, Livingston M, Woods K, Fischbacher M, MacDonald E. Multidisciplinary perspectives on core networking skills. A study of skills: and associated training needs, for professionals working in managed clinical networks. J Interprof Care. 2005;19(2):156-63.

32. Otte IC, Jung C, Bally K, Elger BS, Schildmann J. Interprofessional Silence at the End of Life: Do Swiss General Practitioners and Hospital Physicians Sufficiently Share Information About Their Patients? Journal of palliative medicine. 2016;19(9):983-6.

33. Perley CM. Physician use of the curbside consultation to address information needs: report on a collective case study. Journal of the Medical Library Association. 2006;94(2):137-44.

34. Pinelli V, Stuckey HL, Gonzalo JD. Exploring challenges in the patient's discharge process from the internal medicine service: A qualitative study of patients' and providers' perceptions. J Interprof Care. 2017;31(5):566-74.

35. Politi MC, Pieterse AH, Truant T, Borkhoff C, Jha V, Kuhl L, et al. Interprofessional education about patient decision support in specialty care. Journal of Interprofessional Care. 2011;25(6):416-22.

36. Sampson R, Barbour R, Wilson P. The relationship between GPs and hospital consultants and the implications for patient care: a qualitative study. BMC Fam Pract. 2016;17:45.

37. Shershneva MB, Carnes M, Bakken LL. A model of teaching-learning transactions in generalist-specialist consultations. The Journal of continuing education in the health professions. 2006;26(3):222-9.

38. Sibert L, Lachkar A, Grise P, Charlin B, Lechevallier J, Weber J. Communication between consultants and referring physicians: A qualitative study to define learning and assessment objectives in a specialty residency program. Teaching and Learning in Medicine. 2002;14(1):15-9.

39. Stalhammar J, Holmberg L, Svardsudd K, Tibblin G. Written communication from specialists to general practitioners in cancer care. What are the expectations and how are they met? Scandinavian Journal of Primary Health Care. 2009;16(3):154-9.

40. Sunderji N, Waddell A, Gupta M, Soklaridis S, Steinberg R. An expert consensus on core competencies in integrated care for psychiatrists. General Hospital Psychiatry. 2016;41:45-52.

41. Swar S, Rimal P, Gauchan B, Maru D, Yang Y, Acharya B. Delivering Collaborative Care in Rural Settings: Integrating Remote Teleconsultation and Local Supervision in Rural Nepal. Psychiatric services (Washington, DC). 2019;70(1):78-81.

42. Vargas I, Garcia-Subirats I, Mogollon-Perez AS, Ferreira-de-Medeiros-Mendes M, Eguiguren P, Cisneros AI, et al. Understanding communication breakdown in the outpatient referral process in Latin America: a cross-sectional study on the use of clinical correspondence in public healthcare networks of six countries. Health policy and planning. 2018;33(4):494-504.

43. Wadhwa A, Lingard L. A qualitative study examining tensions in interdoctor telephone consultations. Medical education. 2006;40(8):759-67.

44. Westerman RF, Hull FM, Bezemer PD, Gort G. A study of communication between general practitioners and specialists. British Journal of General Practice. 1990;40(340):445-9.
