# Supplementary material for: Competencies to promote collaboration between primary and secondary care doctors: an integrative review
Source: BMC Fam Pract. 2020 Sep 2;21:179. doi: 10.1186/s12875-020-01234-6 (PMC7469099; doi:10.1186/s12875-020-01234-6)
Supplement: Supplementary file 3 — Additional file 3. Critical appraisal included articles. [file 12875_2020_1234_MOESM3_ESM.docx]

Additional file 3: Critical appraisal

| **Author** | **Method** | **Year** | **Was the sample used in the study appropriate to its research question?** | **Were the data collected appropriately?** | **Were the data analysed appropriately?** | **Can I transfer the results of this study to my own setting?** | **Does the study adequately address potential ethical issues, including reflexivity?** | **Overall: is what the researchers did clear?** | **Critical Appraisal Score (CAS)** |
| --- | --- | --- | --- | --- | --- | --- | --- | --- | --- |
| Beaser | Questionnaires | 2011 | partly | partly | yes | partly | no | yes | 7/12 |
| Beaulieu | Interviews: individual and groups interviews  Review of programs | 2009 | yes | yes | yes | yes | partly | yes | 11/12 |
| Berendsen | Semi-structured interviews. | 2007 | yes | yes | yes | yes | partly | yes | 11/12 |
| Berendsen | Semi-structured interviews | 2006 | yes | yes | yes | yes | partly | yes | 11/12 |
| Bethell | Questionnaires | 2015 | partly | yes | partly | partly | partly | yes | 8/12 |
| Boulware | Survey | 2009 | partly | yes | yes | partly | yes | yes | 10/12 |
| Chong | In depth semi-structured interviews | 2013 | yes | yes | yes | yes | yes | yes | 12/12 |
| Desmedt | Questionnaires and mirror questionnaires | 2002 | partly | yes | partly | partly | no | partly | 6/12 |
| Doohan | Reflection paper | 2017 | na | na | na | partly | na | yes | No score |
| Dossett | Review | 2017 | yes | yes | yes | yes | partly | yes | 11/12 |
| Eyers | Survey | 1994 | yes | yes | partly | partly | no | partly | 7/12 |
| Farnan | Description of a case scenario | 2006 | na | na | na | partly | no | yes | No score |
| Fickel | Semi-structured interviews | 2007 | yes | partly | partly | yes | no | yes | 8/12 |
| Firn | Literature review | 2015 | yes | yes | yes | yes | partly | yes | 11/12 |
| Fleury | Interviews Survey | 2012 | yes | partly | partly | partly | partly | partly | 7/12 |
| Foster | Focus groups Interviews | 2016 | yes | partly | yes | yes | partly | yes | 10/12 |
| Fredheim | Focus groups | 2011 | yes | partly | yes | yes | partly | yes | 10/12 |
| Greer | Interviews | 2015 | yes | yes | yes | yes | partly | yes | 11/12 |
| Grol | Assessment of the letters. Referral letters by 2 assesors, reply letters by 3 assesors with quality criteria based on literature | 2003 | partly | yes | partly | yes | yes | yes | 10/12 |
| van Hasselt | Modified Delphi, 3 rounds | 2015 | yes | yes | yes | yes | partly | yes | 11/12 |
| Hayes | Focus groups  Interviews Survey | 2012 | partly | partly | partly | partly | partly | partly | 6/12 |
| Hysong | Interviews Focus groups | 2011 | yes | yes | yes | yes | no | yes | 10/12 |
| Janssen | Focus groups | 2017 | partly | yes | yes | yes | partly | yes | 10/12 |
| Kessler | Literature review Expert opinions | 2013 | na | na | na | partly | na | yes | No score |
| Kvamme | Recommendations from a work group | 2001 | na | na | na | partly | na | partly | No score |
| Langley | Interviews  Ranking of answers in order of importance | 1989 | partly | partly | partly | yes | no | partly | 6/12 |
| Marshall | Interviews  Focus groups | 1998 | yes | yes | yes | yes | partly | yes | 11/12 |
| Marshall | Interviews  Focus groups | 1998 | yes | yes | yes | yes | partly | yes | 11/12 |
| Mertala | Questionnaires -Open ended -Combined graphical two-dimensional (dimensies: important en implementation) | 2009 | partly | partly | partly | yes | no | partly | 6/12 |
| Newton | Interview | 1993 | partly | partly | yes | partly | no | yes | 7/12 |
| Norris | Focus group Questionnaires based on focus groups Post questionnaire interviews | 2005 | partly | partly | no | partly | no | partly | 4/12 |
| Otte | Interviews | 2016 | yes | partly | yes | yes | partly | yes | 10/12 |
| Perley | Literature review Observation Interviews | 2006 | yes | yes | partly | yes | partly | yes | 10/12 |
| Pinelli | Interviews  Focus groups | 2017 | partly | partly | yes | yes | partly | yes | 9/12 |
| Politi | Review/expert opinion | 2011 | na | na | na | partly | na | yes | No score |
| Sampson | Interviews | 2016 | yes | yes | yes | yes | partly | yes | 11/12 |
| Shershneva | Semi-structured interviews | 2006 | yes | yes | yes | yes | partly | yes | 11/12 |
| Sibert | Literature review  Focus groups | 2002 | yes | partly | no | partly | no | partly | 5/12 |
| Stalhammar | Questionnaires | 2009 | partly | partly | yes | yes | no | yes | 8/12 |
| Sunderji | Interviews  Modified delphi method | 2016 | yes | yes | partly | yes | no | yes | 9/12 |
| Swar | Description  Opinion | 2019 | na | na | na | partly | na | partly | No score |
| Vargas | Questionnaires | 2018 | yes | partly | partly | yes | partly | partly | 8/12 |
| Wadhwa | Documentation  Field observation Semi-structured interviews | 2006 | yes | yes | yes | yes | yes | yes | 12/12 |
| Westerman | Assesment of letters by judges (4 GPs and 4 specialists) based on previous set criteria. | 1990 | partly | yes | yes | partly | no | yes | 8/12 |
|  |  |  |  |  |  |  |  |  |  |

na=not applicable
