# Supplementary material for: Competencies to promote collaboration between primary and secondary care doctors: an integrative review
Source: BMC Fam Pract. 2020 Sep 2;21:179. doi: 10.1186/s12875-020-01234-6 (PMC7469099; doi:10.1186/s12875-020-01234-6)
Supplement: Supplementary file 4 — Additional file 4. Enhancing transparency in reporting the synthesis of qualitative research: the ENTREQ statement. [file 12875_2020_1234_MOESM4_ESM.docx]

**Enhancing transparency in reporting the synthesis of qualitative research: the ENTREQ statement**

| **No Item guide** | **Description** | **In review** |
| --- | --- | --- |
| 1 Aim | State the research question the synthesis addresses. | Described in the introduction. |
| 2 Synthesis  methodology | Identify the synthesis methodology or theoretical framework which underpins the synthesis, and describe the rationale  for choice of methodology (e.g. meta-ethnography, thematic synthesis, critical interpretive synthesis, grounded theory  synthesis, realist synthesis, meta-aggregation, meta-study, framework synthesis). | Described in the methods section, first paragraph. |
| 3 Approach to  searching | Indicate whether the search was pre-planned (comprehensive search strategies to seek all available studies) or iterative (to  seek all available concepts until they theoretical saturation is achieved). | A pre-planned search described in the methods section, paragraph search strategy. |
| 4 Inclusion criteria | Specify the inclusion/exclusion criteria (e.g. in terms of population, language, year limits, type of publication, study type). | Described in the methods section, paragraph search strategy and paragraph inclusion. And some parts in appendix 1. |
| 5 Data sources | Describe the information sources used (e.g. electronic databases (MEDLINE, EMBASE, CINAHL, psycINFO, Econlit), grey  literature databases (digital thesis, policy reports), relevant organisational websites, experts, information specialists, generic web  searches (Google Scholar) hand searching, reference lists) and when the searches conducted; provide the rationale for using  the data sources. | Described in the methods section, paragraph search strategy. |
| 6 Electronic Search strategy | Describe the literature search (e.g. provide electronic search strategies with population terms, clinical or health topic terms,  experiential or social phenomena related terms, filters for qualitative research, and search limits). | Described in the methods section, paragraph search strategy and in appendix 1. |
| 7 Study screening  methods | Describe the process of study screening and sifting (e.g. title, abstract and full text review, number of independent reviewers  who screened studies). | Described in the methods section, paragraph inclusion. |
| 8 Study characteristics | Present the characteristics of the included studies (e.g. year of publication, country, population, number of participants, data  collection, methodology, analysis, research questions). | Described in the results section, mainly table 2. |
| 9 Study selection  results | Identify the number of studies screened and provide reasons for study exclusion (e,g, for comprehensive searching, provide  numbers of studies screened and reasons for exclusion indicated in a figure/flowchart; for iterative searching describe reasons  for study exclusion and inclusion based on modifications t the research question and/or contribution to theory development). | Described in the methods section, paragraph search strategy, paragraph inclusion an critical appraisal. Shown in a Prisma flowchart (figure 2) |
| 10 Rationale for  appraisal | Describe the rationale and approach used to appraise the included studies or selected findings (e.g. assessment of conduct  (validity and robustness), assessment of reporting (transparency), assessment of content and utility of the findings). | Described in the methods section, paragraph critical appraisal. |
| 11 Appraisal items | State the tools, frameworks and criteria used to appraise the studies or selected findings (e.g. Existing tools: CASP, QARI,  COREQ, Mays and Pope [25]; reviewer developed tools; describe the domains assessed: research team, study design, data  analysis and interpretations, reporting). | Described in the methods section, paragraph critical appraisal. |
| 12 Appraisal process | Indicate whether the appraisal was conducted independently by more than one reviewer and if consensus was required. | Described in the methods section, paragraph critical appraisal. |
| 13 Appraisal results | Present results of the quality assessment and indicate which articles, if any, were weighted/excluded based on the  assessment and give the rationale. | Described in the methods section, paragraph critical appraisal and in the results section, mainly table 3. |
| 14 Data extraction | Indicate which sections of the primary studies were analysed and how were the data extracted from the primary studies?  (e.g. all text under the headings “results /conclusions” were extracted electronically and entered into a computer software). | Described in the methods section, paragraph analysis. |
| 15 Software | State the computer software used, if any. | Described in the methods section, paragraph analysis. |
| 16 Number of  reviewers | Identify who was involved in coding and analysis. | Described in the methods section, paragraph analysis and shown in figure 1. |
| 17 Coding | Describe the process for coding of data (e.g. line by line coding to search for concepts). | Described in the methods section, paragraph analysis. |
| 18 Study comparison | Describe how were comparisons made within and across studies (e.g. subsequent studies were coded into pre-existing  concepts, and new concepts were created when deemed necessary). | Described in the methods section, paragraph analysis. |
| 19 Derivation of  themes | Explain whether the process of deriving the themes or constructs was inductive or deductive. | Described in the methods section, paragraph analysis. |
| 20 Quotations | Provide quotations from the primary studies to illustrate themes/constructs, and identify whether the quotations were  participant quotations or the author’s interpretation. | Described in the results section. We stated that all but one quotations are authors’ opinions. |
| 21 Synthesis output | Present rich, compelling and useful results that go beyond a summary of the primary studies (e.g. new interpretation,  models of evidence, conceptual models, analytical framework, development of a new theory or construct). | Described in our result and discussion section. As described in our review, for answering our research question it was necessary to go beyond the results in the primary studies and make new interpretations. |

Tong et al.: Enhancing transparency in reporting the synthesis of qualitative research: ENTREQ. BMC Medical Research Methodology 2012 12:181.
